# Co-assembly of Block Copolymers and Cobalt Ferrite Nanoparticles for Magnetic Material Design

**DOI:** 10.1021/acs.chemmater.5c01442

**Published:** 2025-07-10

**Authors:** Simone Bertucci, Andrea Escher, Gianluca Bravetti, Jean Pierre Miranda Murillo, Gianluca Mazzotta, Sawssen Slimani, Stefano Alberti, Paola Lova, Davide Comoretto, Ullrich Steiner, Davide Peddis, Andrea Dodero

**Affiliations:** † Adolphe Merkle Institute, 311305 University of Fribourg, Chemin des Verdiers 4, 1700 Fribourg, Switzerland; ‡ Department of Chemistry and Industrial Chemistry, University of Genoa, Via Dodecaneso 31, 16146 Genoa, Italy; § Department of Chemistry, University of Fribourg, Chemin du Musée 9, 1700 Fribourg, Switzerland; ∥ National Center of Competence in Research (NCCR) Bio-Inspired Materials, Chemin des Verdiers 4, 1700 Fribourg, Switzerland; ⊥ INSTM RU, nM2-Lab, University of Genoa, Via Dodecaneso 31, 16146 Genova, Italy; # nM2-Lab, CNR − Institute of Structure of Matter, 00015 Rome, Italy

## Abstract

Hybrid materials that integrate photonic and magnetic functionalities are a major focus of next-generation nanotechnology, but their scalable production remains a significant challenge. Here, we present a facile strategy to produce hybrid photonic microparticles by coassembling poly­(styrene)-*b*-poly­(2-vinylpyridine) (PS-P2VP) block copolymers with 10 nm cobalt ferrite nanoparticles within emulsion droplets. This method allows the formation of highly ordered, hierarchical, onion-like structures with alternating concentric layers. Selective localization of the nanoparticles within P2VP domains preserves the periodicity essential for structural coloration while introducing tunable magnetic properties. Optical characterization confirms that the microparticles exhibit a vivid blue structural color and maintain a well-defined photonic bandgap up to a critical nanoparticle concentration, after which the structural order is disrupted. Remarkably, the nanostructure order of the polymer matrix induces a partial alignment of the magnetic easy axis of the nanoparticles, increasing the thermal stability of the magnetization (i.e., increase in the reduced remanent magnetization). This distinctive synergy between photonic and magnetic properties establishes a platform for multifunctional materials with potential applications in magnetically tunable photonic devices, advanced sensors, and responsive materials. The results demonstrate a scalable and versatile approach to fusing photonic architectures with functional nanomaterials, providing design opportunities for next-generation hybrid materials.

## Graphical abstract

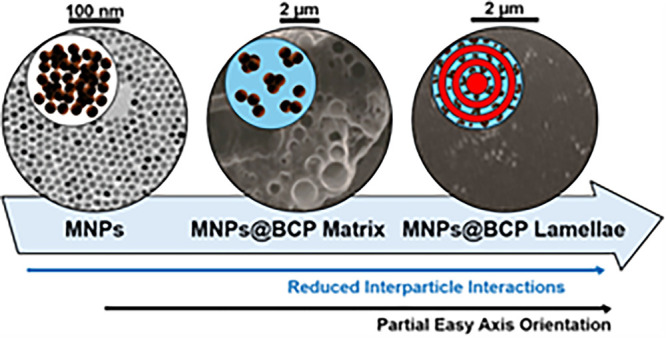

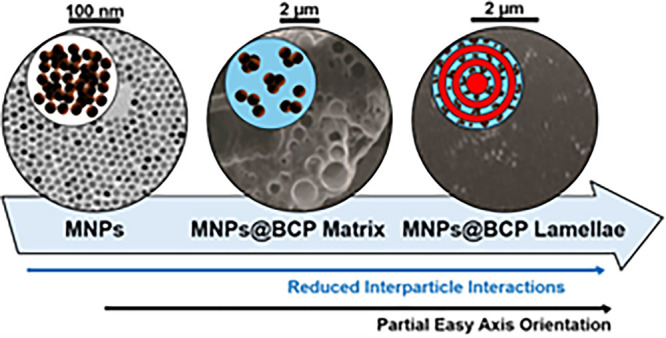

## Introduction

Hybrid materials, combining two or more distinct components synergistically integrated at the nanoscale, have emerged as a cornerstone of advanced materials science in recent years.^1,2^ Their unique properties and multifunctionality hold promise for various applications, such as technological devices, biomedical products, and food packaging.^3–5^

Among the various possible strategies for creating nanocomposite materials, block copolymers (BCPs) stand out as a versatile and powerful platform due to their ubiquitous ability to self-assemble into well-defined nanostructures. This property makes them particularly attractive for developing advanced materials with precise architectures, and their extraordinary structural and compositional versatility has led to groundbreaking applications in several fields.^6–8^ Block copolymers comprise at least two chemically distinct and immiscible monomer units grouped in discrete, covalently linked blocks along the polymer chain and configured into linear, branched, or cyclic molecular architectures. While covalent bonding prevents macrophase separation, favorable interactions between identical blocks favor the formation of well-organized nanostructures that can be manipulated by controlling molecular parameters such as the Flory–Huggins interaction parameter (χ), degree of polymerization (*N*), and block volume fraction (*f*).^9^ Various morphologies have been revealed experimentally and theoretically, including spheres, cylinders, lamellae, etc.^10^

Although the self-assembly in bulk and solution has been extensively studied and is well understood, the confinement of block copolymer self-assembly in emulsion droplets has recently become a paradigm for preparing polymeric nano- and microparticles with unconventional morphologies.^11,12^ In fact, three-dimensional (3D) spherical confinement of BCPs is of particular interest because fundamental variations in morphological ordering are observed due to enhanced interfacial interactions, symmetry breaking, structural frustration, and confinement-induced entropy losses. This particular confinement allows for a wide variety of nanostructures.^13,14^ Such an approach has been successfully explored to fabricate photonic particles consisting of alternating concentric lamellae of two polymer blocks with different refractive indices, whose interaction with light is responsible for a tunable and vibrant structural coloration.^15–19^

While these results are auspicious for developing long-lasting photonic paints, they have also paved the way for exploring advanced materials. In fact, the combination of self-assembled block copolymer nanostructures and inorganic nanoparticles (NPs) has proven to be a straightforward and powerful strategy for fabricating hybrid functional materials with improved optical, electrical, and mechanical properties.^20,21^ The main advantages include preventing nanoparticle aggregation and exerting precise spatial control over their location by fine-tuning the enthalpic and entropic contributions.^22,23^ This can be achieved by manipulating the surface chemistry of the NPs with organic ligands that present functional groups capable of selectively binding to one of the polymeric blocks or with short, wet-brush homopolymer chains that can interpenetrate into the corresponding domains without macrophase separation. Thus, nanostructured block copolymer assemblies can be obtained in which the nanoparticles are located in only one of the blocks or at their interfaces. However, despite several studies that have validated such a strategy in films and microparticles, little work has been done on functional materials that combine photonic structures based on block copolymers and NPs.^20,24,25^ The main limitation stems from the domain periodicity required to ensure a photonic bandgap (PBG) in the visible, which can only be achieved with high molecular weight block copolymers.^26^

Here, we design hybrid block copolymer photonic microparticles consisting of alternating concentric layers in which one of the phases is selectively loaded with magnetic cobalt ferrite nanoparticles. Cobalt ferrite (CoFe_2_O_4_) nanoparticles are known for their peculiar magnetic properties, such as high coercivity and moderate saturation magnetization.^27^ Additionally, these nanoparticles exhibit significant chemical stability and mechanical hardness, and their magneto-optical properties enable their use in advanced photonic and spintronic devices, highlighting their versatility and importance in modern nanotechnology.^28,29^ The hybrid photonic microparticles are fabricated via a solvent evaporation process (Figure 1) by mixing *ex-situ* synthesized magnetic nanoparticles (MNPs) with poly­(styrene)-*b*-poly­(2-vinylpyridine) (PS-P2VP) block copolymer in chloroform and emulsifying the mixture with a continuous aqueous phase containing poly­(vinyl alcohol) (PVA) as a surfactant. Preferential interactions between the nanoparticles and the block copolymer ensure selective segregation into P2VP layers^30–32^ resulting in a well-ordered onion-like structure giving rise to an intense blue structural coloration. Our results demonstrate that it is possible to preserve precise periodic architectures, which are essential for photonic applications, while embedding nanoparticles that impart magnetic properties to the resulting nanostructured material. This dual functionality opens up several possibilities for advanced materials with great potential for applications in optoelectronics and magneto-optical devices.

**Figure 1:**
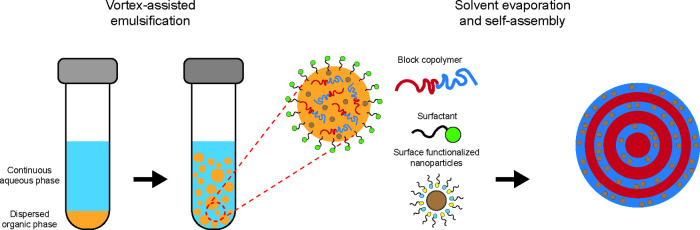
Schematic representation of the confined coassembly process leading to the formation of hybrid photonic microparticles. The PS-P2VP diblock copolymer and cobalt ferrite nanoparticles are dispersed in chloroform and emulsified in an aqueous phase containing PVA as a stabilizer. Upon evaporation of chloroform, the block copolymer self-assembles into an onion-like lamellar structure, with the MNPs selectively segregating within the P2VP domains due to favorable interactions. The resulting microparticles exhibit a well-ordered concentric morphology, producing structural coloration due to the periodic refractive index contrast between the polymeric domains while imparting magnetic functionality.

## Experimental Section

### Materials

Poly­(styrene)-*b*-poly­(2-vinylpyridine) with *M*_n_PS_ = 185 kg/mol, *M*_n_P2VP_ = 195 kg/mol and a polydispersity index (PDI) of 1.05 was purchased from Polymer Source and used without purification. Poly­(vinyl alcohol) (PVA) with *M*_w_ = 13 – 23 kg/mol and a degree of hydrolysis of 87 – 89%, chloroform (CHCl_3_) (containing 100 – 200 ppm amylene as stabilizer, ≥ 99.5%), tris­(acetylacetonate) iron­(III) (Fe­(acac)_3_) (99%), cobalt­(II) acetylacetonate (Co­(acac)_2_) (97%), oleic acid (90%), oleylamine (70%), and 1,2-hexadecanediol (90%) were purchased from Sigma-Aldrich.

### Methods

#### Synthesis of Cobalt Ferrite Nanoparticles

The synthesis of cobalt ferrite (CoFe_2_O_4_) nanoparticles was performed via a thermal decomposition method.^33^ Briefly, 2 mmol Fe­(acac)_3_, 1.29 mmol Co­(acac)_2_, 6 mmol oleic acid, 6 mmol oleylamine, and 10 mmol 1,2-hexadecanediol were mixed in 20 mL of benzyl ether in a three-necked balloon. The resulting suspension was heated to 270 °C under magnetic stirring using a heating mantle connected to a Proportional Integral Derivative (P.I.D.) apparatus for precise temperature control. In the first step, the reaction mixture was degassed under vacuum at 110 °C for 60 min and then heated at 200 °C for 120 min. A change in the color of the mixture to dark black was observed as an indication of the formation of nanoparticles,^34^ and the temperature was then increased to 270 °C for 60 min for particle growth. Three washing cycles were performed by centrifugation (5 min, 5000 rpm), adding fresh EtOH each time to remove the supernatant. Finally, the magnetic precipitate was redispersed in toluene, and CoFe_2_O_4_ ferrofluid was obtained.

#### Preparation of Hybrid Photonic Microparticles

PS-P2VP was solubilized in CHCl_3_ at a concentration of 10 mg/mL with stirring at room temperature for a few hours. PVA was solubilized in Milli-Q at a concentration of 10 or 3 mg/mL with stirring at *T* = 85 °C for 2 h. In a typical experiment, 0.25 mL of BCP solution in CHCl_3_ was mixed with various volumes of MNP suspension in toluene (12 mg/mL), as detailed in Table S1. The mixtures were then dried under vacuum at *T* = 40 °C, and the residual solid was redispersed in 0.25 mL of CHCl_3_. The resulting suspension was then emulsified with 2.5 mL of PVA solution (10 mg/mL) using laboratory vortex equipment. The emulsification procedure was performed in a 7 mL screw cap at a speed of 2000 rpm for 30 s. The resulting oil-in-water (O/W) emulsion was collected in a covered 5 cm Petri dish containing 15 mL of PVA solution (3 mg/mL) and left to dry for 2 days. The resulting solid microparticles were collected in a 50 mL plastic falcon, centrifuged at 10000 rpm for 10 min, and washed at least three times with 15 mL of Milli-Q to remove any residual surfactant before finally dispersing in 2 mL Milli-Q water.

#### Characterization Techniques

Cobalt ferrite nanoparticles were first characterized by X-ray diffraction (XRD) using a Seifert 3003 TT diffractometer equipped with a secondary graphite monochromator, using Cu *K*_*α*_ radiation (λ = 1.5418 AÅ). Measurements were performed in the 2θ range from 20 to 90° with a step size of 0.01°, counting 1 s per step.

Transmission electron microscopy (TEM) observations were performed using a Philips CM200 microscope operating at 200 kV and equipped with a LaB_6_ filament. For TEM analysis of the nanoparticles, samples were prepared as a suspension by adding the sample to ethanol (1 mg/mL). For TEM analysis of the block copolymer–nanoparticle dispersion, samples were prepared in chloroform. A drop of the mixture was deposited on a carbon-coated TEM grid and kept in air until the solvent was completely evaporated.

UV–vis spectroscopy was performed on 1 mg/mL nanoparticle suspension in toluene using a Shimadzu UV-2401 PC spectrometer. The same suspension was characterized by photoluminescence (PL) spectroscopy using a Horiba Fluorolog 3 spectrometer with an excitation wavelength of 355 nm. The light source was a 450 W xenon lamp, and the detector was an FL-1030-UP photomultiplier.

Thermogravimetric analysis (TGA) was performed using a Mettler-Toledo TGA/DSC 1 STARe system. Degradation profiles were studied under a nitrogen flow of 80 mL/min in the temperature range of 30 – 750 °C at a heating rate of 10 °C/min.

The hybrid photonic microparticles were characterized via optical microscopy using a customized microscope (ZEISS Axio Scope.A1) equipped with a CCD camera (Point Gray GS3-U3–28S5C–C) calibrated against a standard white diffuser and a halogen lamp as a light source. Micrographs were taken in reflection mode in a bright field configuration using a 50× objective (Zeiss LD EC Epiplan-Neofluar, NA = 0.8). The reflectance spectra of the microparticles were measured by microspectroscopy by coupling the above-described microscope to a diode array spectrometer (Ocean Optics QEPro) using an optical fiber confocally positioned at the image plane of the microscope (Avantes QP230–2-XSR, 230 μm core size). An aluminum mirror (Thorlabs PF10–03-G01) was used as a reference. Photonic microparticles were characterized in water suspensions on optical glass slides covered with a glass coverslip.

The internal morphology of the hybrid microparticles was investigated by focused ion beam scanning electron microscopy (FIB-SEM) using a Thermo Scientific Scios 2 DualBeam FIB-SEM (FEI, Eindhoven, The Netherlands). The samples were coated with a 4 nm thick gold layer to ensure good conductivity. Particles were milled using a Ga^+^ ion beam with an acceleration voltage of 30 kV and a current of up to 3 nA. The cross sections were imaged with the integrated SEM Everhart-Thornley (ETD, secondary electrons) and in-lens (T1, A+B composite mode, backscattered electrons) detectors set to a voltage of 5 kV and a current of 0.4 nA. PS-P2VP particles were stained with an aqueous iodine solution to enhance the contrast between the BCP domains.

Small-angle X-ray scattering (SAXS) and ultrasmall angle X-ray scattering (USAXS) measurements were performed on the ID02 beamline at the European Synchrotron Radiation Facility (ESRF) in Grenoble. Circular samples with an average thickness of 1 mm and a diameter of 4 mm were prepared by drop-casting particle suspensions in polytetrafluoroethylene (PTFE) disks sealed on both sides with Kapton tape (DuPont).

Magnetic properties were investigated using a commercial Quantum Design Physical Property Measurement System (PPMS). Powder samples were fixed in polycarbonate capsules with epoxy resin to prevent the particles from moving during the measurements. Magnetization was measured as a function of the applied magnetic field at 5 and 300 K, and all measurements were normalized to the weight of the magnetic phase. Temperature-dependent magnetization was studied using zero-field-cooled (ZFC) and field-cooled (FC) protocols. For the ZFC measurements, the samples were first cooled from 300 to 5 K in a zero magnetic field, then a small field (2.5 mT) was applied, and *M*_ZFC_ was measured during the warm-up. For FC measurements, the samples were cooled while maintaining the applied field, and *M*_FC_ was measured as the temperature increased.

In addition, the field dependence of the remanent magnetization was measured using remanence techniques, namely IRM (isothermal remanent magnetization) and DCD (direct current demagnetization) protocols. In the IRM protocol, starting from a demagnetized sample, a small positive magnetic field (*H*_rev_) was applied and then removed, and the remanence *M*_IRM_ was recorded in a zero field. This step was repeated by increasing the applied field until saturation was reached. For DCD measurements, a sample was first saturated, then a small magnetic field opposite to the saturation direction was applied for 90 s, turned off, and the remanence *M*_DCD_ was measured. This protocol was then repeated by increasing the applied field in the opposite direction until remanence saturation was reached.

## Results & Discussion

The morpho-structural features of the magnetic nanoparticles were investigated by X-ray powder diffraction (XRD) and transmission electron microscopy (TEM). The XRD pattern (Figure 2a) indicates the presence of a cubic spinel structure, and the crystallite size is estimated to be about 8.3(4) nm using the Scherrer equation.

**Figure 2:**
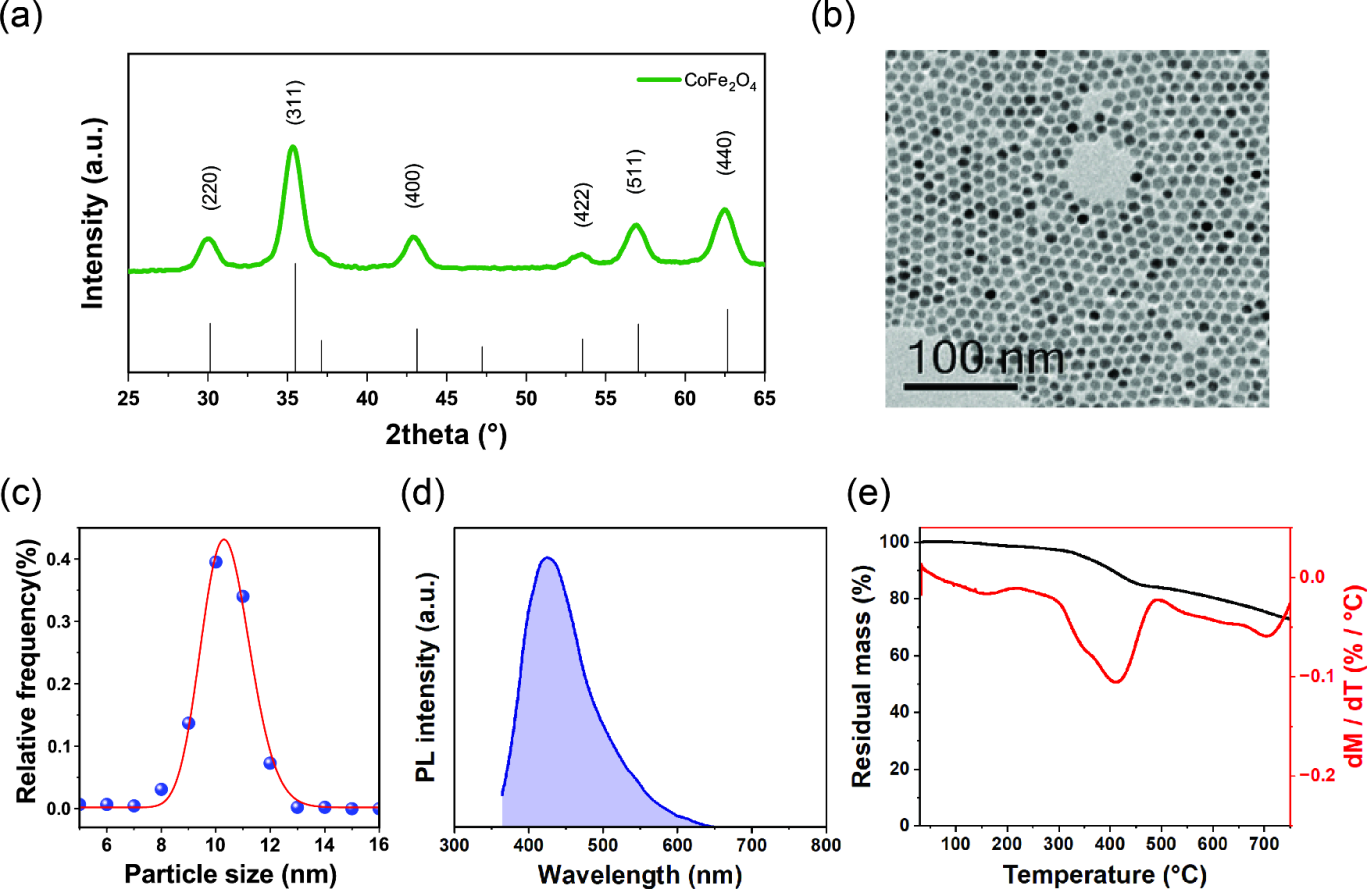
(a) X-ray diffraction (XRD) pattern of CoFe_2_O_4_ nanoparticles. Vertical bars correspond to the standard reference pattern for spinel CoFe_2_O_4_ (JCPDS card No. 03-0864). (b) TEM micrograph of CoFe_2_O_4_ nanoparticles showing a relatively homogeneous spherical shape with (c) size distribution of 10(1) nm (value in parentheses indicates the standard error (±0.1 nm) on the fitted average size). (d) PL spectrum of MNPs dispersed in toluene at 1 mg/mL concentration with a distinct emission peak detected at 423 nm. (e) TGA and DTG curves of MNPs measured in a nitrogen atmosphere (80 mL/min) at a heating rate of 10 °C/min. Three main degradation processes are observed: (i) at *T* **<** 200 °C corresponding to the removal of residual moisture and bound water, (ii) from 300 to 500 °C corresponding to the carbonization of the organic ligands on the particle surface, and (iii) from 500 to 700 °C corresponding to the decarbonization of the nanoparticle surface.

TEM analysis further confirms the formation of nanoparticles. As shown in Figure 2b and Figure 2c monodispersed spherical cobalt ferrite nanoparticles are obtained with an average size of 10(1) nm. These MNPs are ideal for integration into photonic structures because their size is sufficiently small to strongly limit light scattering, thus preserving the integrity and performance of the optical properties.^35,36^ In addition, the CoFe_2_O_4_ nanoparticles are superparamagnetic (i.e., zero remanence magnetization and zero coercive field) at 300 K, making them ideal for external manipulation using a magnetic field.^37^ Furthermore, one of the most critical factors affecting both the assembled morphology of BCP/NP hybrid systems and the spatial distribution of NPs is the size ratio between NPs (*d*) and their associated domain (*L*). It has been shown that when *d/L* < 0.2, nanoparticles can be homogeneously distributed within a BCP structure without phase segregation occurring.^38–40^ Assuming *L* is around 70 nm based on our previous work,^15^ *d/L* is expected to be close to 0.1, thus meeting the predicted size requirements.

The optical properties of MNPs were also investigated by photoluminescence (PL) and UV–vis spectroscopy. The PL spectrum of the nanoparticles dispersed in toluene (Figure 2c) shows a broad visible emission signal, which is attributed to the charge transfer between Fe^3+^ at both tetrahedral and octahedral sites and Co^2+^ at octahedral sites,^41^ and a narrow emission peak centered at 423 nm, which is attributed to the recombination of photoinduced electrons and holes in the conduction and valence bands.^42^ At the same time, the UV–vis spectrum (Figure S1) shows a broadband absorption in the visible and UV region, which is consistent with the dark color of cobalt ferrite nanoparticles.

TGA and DTG curves measured in a nitrogen atmosphere are shown in Figure 2d, where three main weight losses are observed.^43^ The first, occurring below 200 °C, corresponds to the dehydration of absorbed moisture. The second, occurring in the temperature range of 200 – 450 °C, is attributed to the carbonization of the ligands (i.e., oleic acid and oleylamine) adsorbed on the nanoparticle surface. The third and final one, from 450 to 700 °C, is attributed to the final decarbonization due to the nitrogen atmosphere. A total percentage weight loss of 25% is observed in the investigated temperature range, indicating that a considerable amount of oleic acid and oleylamine molecules are adsorbed on the nanoparticle surface. Remarkably, such ligands provide direct control over the enthalpic and entropic interactions between the block copolymer chains and the MNPs, a fundamental requirement in the coassembly strategy.^21^

Following this assumption, hybrid microparticles were prepared in a one-step process by confining the self-assembly of poly­(styrene)-*b*-poly­(2-vinylpyridine) and the synthesized cobalt ferrite MNPs in emulsion droplets. At first, the optimal dispersion of the nanoparticles in the block copolymer solution was evaluated via TEM analysis, with the results reported in Figure S2. Concerning the self-assembly process, in the absence of MNPs, the slow diffusion of chloroform into the aqueous phase allows the BCP chains to phase-separate into a well-ordered morphology, with the preferential interactions between the surfactant (i.e., PVA) and the P2VP block inducing the formation of concentric lamellae.^15,20^ This results in solid microparticles with an intense blue color, as shown in the optical microscopy image in Figure 3a. To evaluate the effect of the addition of nanoparticles on the self-assembled structure and associated optical properties, an increasing amount of the inorganic component is added up to a nominal value of 32.4 wt %. Figure 3a shows the collected optical microscopy images. The presence of MNPs is immediately confirmed by the reddish color adopted by the microparticles, which becomes more intense at higher nanoparticle concentrations.

**Figure 3:**
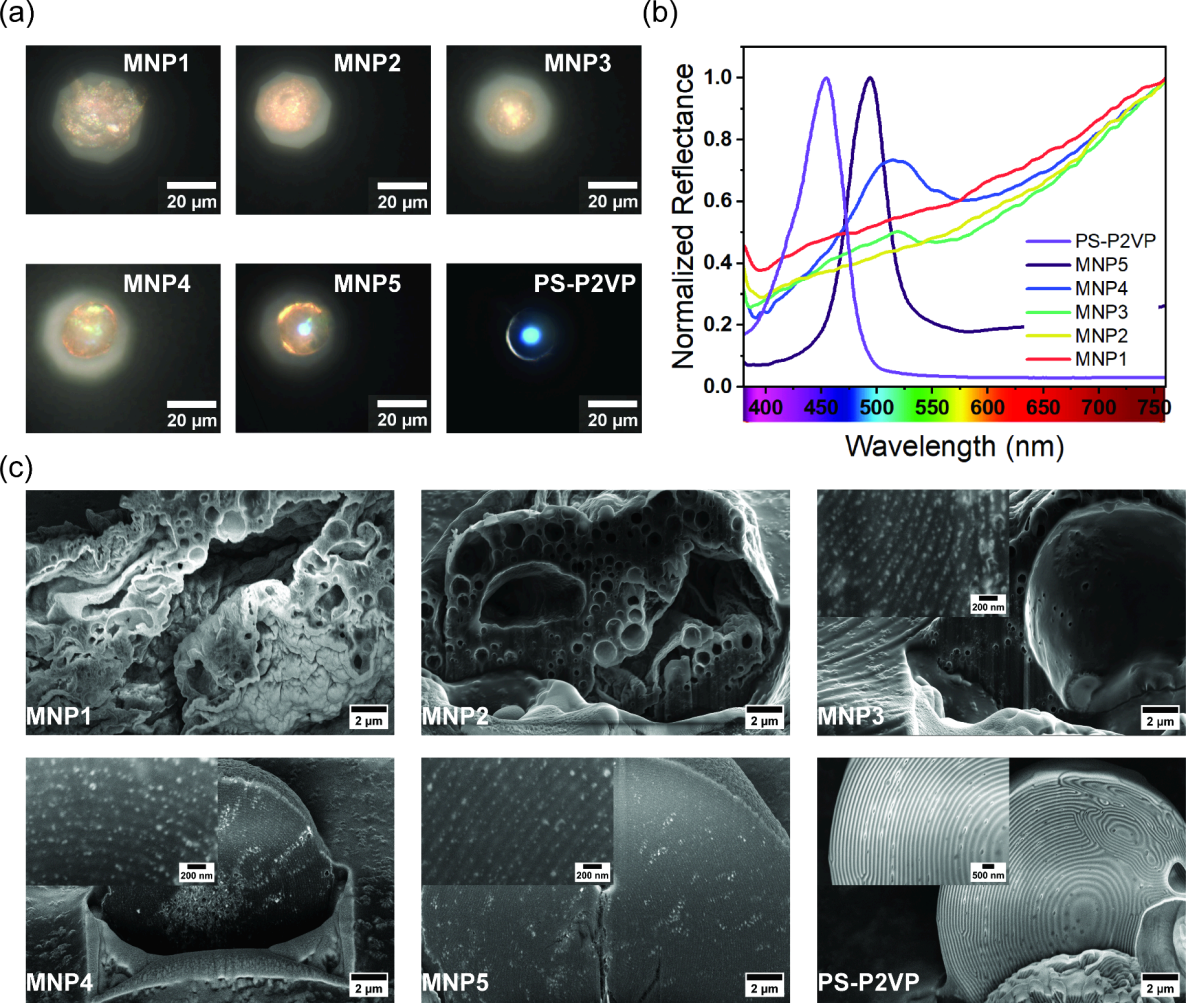
(a) Optical microscopy images of microparticles prepared with increasing CoFe_2_O_4_ nanoparticle content. The structural blue coloration observed in pure PS-P2VP microparticles gradually fades with increasing MNP concentration, resulting in a reddish hue at high loading levels. (b) Normalized reflectance spectra of individual hybrid microparticles showing a distinct photonic reflectance peak for PS-P2VP and MNP5 samples, with a redshift upon nanoparticle incorporation. At high nanoparticle loading (i.e., MNP3, MNP2, and MNP1), the reflectance is dominated by MNP absorption. (c) FIB-SEM cross-sectional images reveal the internal morphology of the microparticles. The concentric lamellar structure is well preserved for PS-P2VP and MNP5, partially disrupted for MNP4, and completely lost at higher loading levels (i.e., MNP3, MNP2, MNP1), where macrophase separation of the nanoparticles occurs. Selective localization of MNPs within P2VP domains is observed at all concentrations.

To further verify the successful incorporation of MNPs into the block copolymer matrix, thermogravimetric analysis was performed in a nitrogen atmosphere on 5 mg of dried microparticles. Figure S3 shows the presence of two different degradation phenomena occurring around 250 °C and in the 350 – 450 °C temperature range. While the first one corresponds to the carbonization of the organic ligands on the particle surface, as already discussed in Figure 2d, the second one is associated with the degradation of the block copolymer chains. An evident increase in the inorganic residue at *T* = 600 °C is observed with increasing mass fraction of added MNPs (Table S1). This result indicates the possibility of effectively loading a high amount of nanoparticles into the block copolymer structure via a simple confined coassembly procedure. It should be noted that the much higher residual mass obtained from TGA measurements compared to the nominal MNP weight ratio is due to the nitrogen atmosphere in which the experiments were conducted.

Interestingly, the optical microscopy images in Figure 3a show a distinct change in the appearance of the microparticles. At high loading concentrations (MNP1), the microparticles lose their sphericity, and their surface becomes rough, as shown in Figure 3a. In addition, the blue structural color is not observed in favor of the reddish color imparted by the nanoparticles. While similar results are found for MNP2 and MNP3, lowering the loading concentration allows the formation of spherical, homogeneous microparticles where both blue and red colors are visible. In particular, MNP5 is characterized by an intense structural coloration localized in the center of the particle, which is then surrounded by a reddish outline. Further insight was provided by reflection microspectroscopy, where spectra measured for single particles are shown in Figure 3b. Consistent with their appearance, PS-P2VP and MNP5 samples show a well-defined reflection peak centered at λ_max_ equal to 450 and 500 nm, respectively. This is due to the ordered photonic structure consisting of concentric lamellae formed by the block copolymer. For MNP4, a much broader and less pronounced reflection peak is observed at λ_max_ = 515 nm, which still indicates the self-assembly of the block copolymer chains into a photonic structure. However, at higher loading concentrations (i.e., MNP3, MNP2, and MNP1), no obvious reflection peaks are detected in the measured spectra, with the overall optical response dominated by the cobalt ferrite nanoparticles.

FIB-SEM was performed to evaluate the internal structure of the fabricated particles, with top-view micrographs shown in Figure S4. As observed by optical microscopy, the addition of high-loading of MNPs reduces the sphericity and homogeneity of the microparticles. Such a finding is observed in Figure 3c, which shows the micrographs of the particle cross sections. While well-defined and homogeneous concentric lamellae are obtained for PS-P2VP and MNP5 samples, and to some extent also for MNP4, the onion-like structure becomes irregular by adding a higher amount of the MNPs. These morphological disruptions are mainly attributed to a nanoparticle-induced alteration in interfacial tension. Oleic acid/oleylamine-functionalized nanoparticles exhibit amphiphilic character and preferentially accumulate at the oil–water interface, acting similarly to colloidal surfactants. At elevated concentrations, this accumulation disrupts the interfacial energy landscape and introduces curvature instabilities, akin to phenomena observed in Pickering emulsions. This results in morphological collapse and loss of long-range order, as seen in FIB-SEM cross sections and reflected in the disappearance of photonic bandgap features (Figure 3b). While magnetic dipole interactions may further contribute, similar instabilities in systems with nonmagnetic nanoparticles support a general interfacial mechanism as the dominant cause. However, although only a limited amount of cobalt ferrite can be effectively added to the block copolymer structure before disrupting the self-assembly, we verify preferential interactions between one of the blocks and the MNPs, as the selective spatial distribution of MNPs within the P2VP layers is observed for all samples, regardless of the loading concentration. This can be explained by the ability of the organic ligands used to stabilize the nanoparticles to control their enthalpic and entropic interactions with the block copolymer chains,^22,23,25^ and it can also be assumed that such interactions allow increasing the fraction of nanoparticles that can be effectively loaded within the BCP structure without macrophase separation.

Structural analysis was also performed via scattering experiments to confirm the FIB-SEM data. Figure S5 shows the USAXS and SAXS spectra, combined to span a wide *q* range, obtained for the fabricated hybrid particles. In all cases, a prominent scattering peak associated with the cobalt ferrite nanoparticles is detected at *q*_MNP_ ∼ 0.7 nm^–1^, corresponding to a size of about 9 nm, in agreement with the TEM (Figure 2b). More interestingly, MNP5 and MNP4 samples show a well-defined peak at *q*_BCP_ values of 0.042 nm^–1^ and 0.041 nm^–1^, giving a domain periodicity of about 149.5 and 153.2 nm, respectively. As expected, no clear scattering signals can be observed for MNP3, MNP2, and MNP1 due to the poor order of the self-assembled structures.

The obtained samples were further investigated by magnetization measurements. The temperature dependence of the magnetization shows that all samples exhibit similar behavior. Figure 4a shows ZFC-FC curves for sample MNP5 as an example (other ZFC-FC curves are shown in Figure S6 for clarity). Typically, in an ideal system of noninteracting nanoparticles of uniform size, the temperature corresponding to the maximum in the ZFC magnetization curve (*T*_max_) corresponds to the blocking temperature (*T*_b_). In real systems, however, nanoparticles have a size distribution that causes the ZFC curve to broaden and *T*_max_ to shift. This shift is described by the relationship *T*_max_ = *βT*_b_, where β (∼ 1.5–2) is a coefficient that depends on the particle size distribution.^44^ *T*_b_ is the temperature at which the relaxation time is equal to the time scale of the experimental technique used. For an ensemble of nanoparticles with a size distribution, *T*_b_ can be defined as the temperature at which 50% of the particles are in the superparamagnetic regime. On the other hand, *T*_b_ is proportional to the magnetic anisotropy energy barrier *E*_a_ (*E*_a_ = *KV*, where *K* is the anisotropy constant and *V* is the particle volume) and can be estimated from the distribution in *E*_a_ as the temperature at which 50% of the particles overcome their anisotropy energy barriers.^45,46^ In all samples studied, *M*_ZFC_ and *M*_FC_ (see experimental section for details) show irreversible behavior below a certain temperature (*T*_irr_ ≈ 260 K), corresponding to the blocking of the largest particles.^47^ Below *T*_irr_, the FC curves for all samples show a temperature-independent magnetization, suggesting the presence of interparticle interactions leading to a frozen magnetic ordered state with high anisotropy.^48^

**Figure 4:**
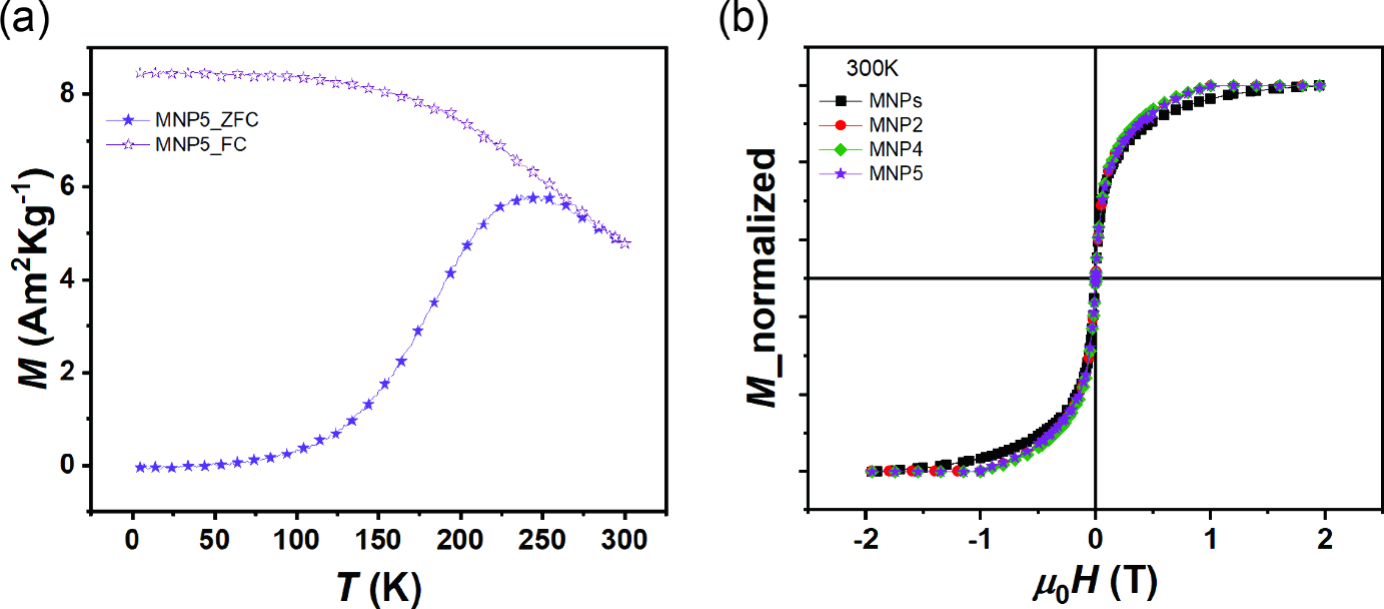
(a) ZFC (full stars) and FC (empty stars) curves for MNP5. (b) Field dependence of magnetization recorded at 300 K for MNPs, MNP2, MNP4, and MNP5.

The field dependence of the magnetization was investigated at 300 K (Figure 4b) and 5 K (Figure 5), and the extracted values of saturation magnetization (*M*_s_), coercive field (*H*_c_), reduced remanent magnetization (*M*_r_/*M*_s_), and anisotropy field (*H*_a_) are listed in Table 1.

**Table 1:** Saturation Magnetization (*M*_s_), Reduced Remanent Magnetization (*M*_r_/*M*_s_), Anisotropy Field (*μ*_0_ *H*_a_), Coercivity (*μ*_0_ *H*_c_), and Remanence Coercivity (*H*_cr_) at 5 K^a^

Sample	*T*_b_ (K)	*T*_irr_ (K)	*M*_s_ (A m^2^ kg^–1^)	*H*_c_ (T)	*M*_r_/*M*_s_	*H*_a_ (T)	*H*_cr_ (T)
MNPs	188	280	96(1)	1.25(2)	0.63	3.7(1)	1.49(2)
MNP2	193	263	95(3)	1.4(2)	0.68	3.8(2)	1.60(1)
MNP4	185	261	62 (9)	1.4(2)	0.74	3.8(1)	1.58(3)
MNP5	183	260	58(1)	1.4(1)	0.79	3.9(1)	1.60(1)

a Uncertainties are given in brackets.

**Figure 5:**
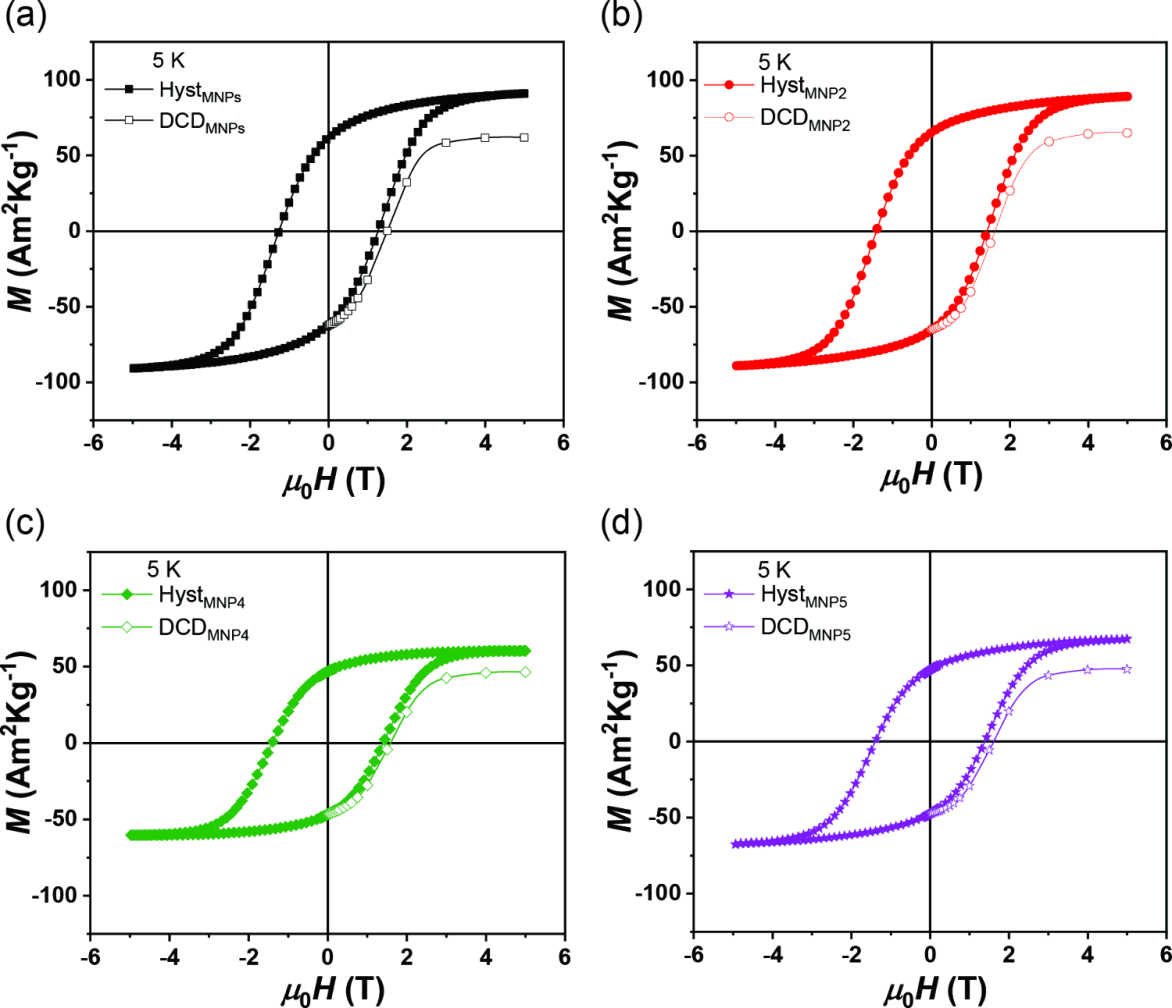
Field dependence of magnetization (full symbols) and DC demagnetization (DCD, empty symbols) curves measured at 5 K for (a) MNPs, (b) MNP2, (c) MNP4, and (d) MNP5.

*H*_a_ is estimated as the point where the difference between the magnetizing and demagnetizing branches of the hysteresis loop, normalized by the saturation magnetization *M*_s_, becomes less than 3%. This threshold indicates the field at which the magnetization response exhibits minimal hysteresis, reflecting near-reversible behavior.^49^ At 300 K, MNPs exhibit superparamagnetic behavior characterized by zero remanence magnetization (*M*_r_) and zero *H*_c_,^50^ which was maintained after embedding in the BCP matrix regardless of concentration. At 5K, the *M*_s_ values of all samples approach those of bulk CoFe_2_O_4_ within the experimental error. Interestingly, the reduced remanent magnetization was similar for MNPs and MNP2, then it increased to 0.8 (i.e., close to the theoretical value of cubic anisotropy)^51^ for MNP4 and MNP5, suggesting that the textural properties of the polymeric matrix can play a significant role in modifying the magnetic properties. The values of *H*_c_ and *H*_a_ decrease from the MNPs sample to the other polymer-embedded samples (from 1.25 to 1.40 T), and appear to be essentially independent of the particle concentration. Figure 5 shows the hysteresis loops (full symbols) and remanent DC demagnetization (DCD) curves (empty symbols) at 5K. In general, only the trapped particles (i.e., particles not in the superparamagnetic regime) contribute to the remanent magnetization, and therefore, *M*_DCD_ can be considered sensitive only to the irreversible component of the magnetization.^52^ The value of the field where *M*_DCD_ is zero, called the remanence coercivity (*H*_cr_, Table 1), also shows an increase after embedding the MNPs in the BCPs matrix. It is interesting to note that the ratio *H*_a_/*H*_c_ is almost equal in bare MNPs (∼ 1.13) and nanoparticles in BCP (∼ 1.15) samples, suggesting that the difference between bare particles and particles embedded in the matrix is not affected by the relaxation process of magnetization.

To better explore the scenario of interparticle interactions, *δm* plots are shown. As an example, Figure 6a shows normalized *m*_r___DCD_(*H*) (full symbols) and *m*_r___IRM_(*H*) (empty symbols) for MNPs (other curves are shown in Figure S7). For an assembly of noninteracting single-domain particles with uniaxial anisotropy and magnetization reversal by coherent rotation, the two remanence curves are related by the Wohlfarth equation,^53,54^

1 $$\delta m=m_{\mathrm{DCD}}\left(H\right)-1+2m_{\mathrm{IRM}}\left(H\right)$$

where *m*_DCD_(*H*) and *m*_IRM_(*H*) are the reduced terms *M*_DCD_(*H*)/*M*_DCD_(5T) and *M*_IRM_(*H*)/M_IRM_(5T), *M*_DCD_(5T) and *M*_IRM_(5T) are the remanence values from the DCD and IRM curves at 5 T, respectively. In general, a negative deviation of dm from the linearity condition indicates the predominance of demagnetizing (i.e., dipole–dipole) interactions, while a positive deviation indicates the predominance of magnetizing (i.e., exchange) interactions. In our case, *δm* plots in Figure 6b show the predominance of dipolar interactions for all samples. For MNPs, *δm* peak is centered at 1.26(2) T, while for MNP2, MNP4, and MNP5 the peak is centered at 1.33(2) T, 1.31(1) T, and 1.34(2) T, respectively. The observed shift to a higher field is accompanied by a decrease in *δm* amplitude (i.e., the strength of the interparticle interactions), indicating that the BCPs matrix plays an important role in tuning the interparticle dipolar interactions (i.e., increasing the interparticle distance). In particular, interparticle interactions in MNPs are about 2.2% higher than in the other samples, which, however, show no significant changes between each other. The overall summary of the results discussed indicates that the textural properties of the matrix influence the magnetic properties of the sample.

**Figure 6:**
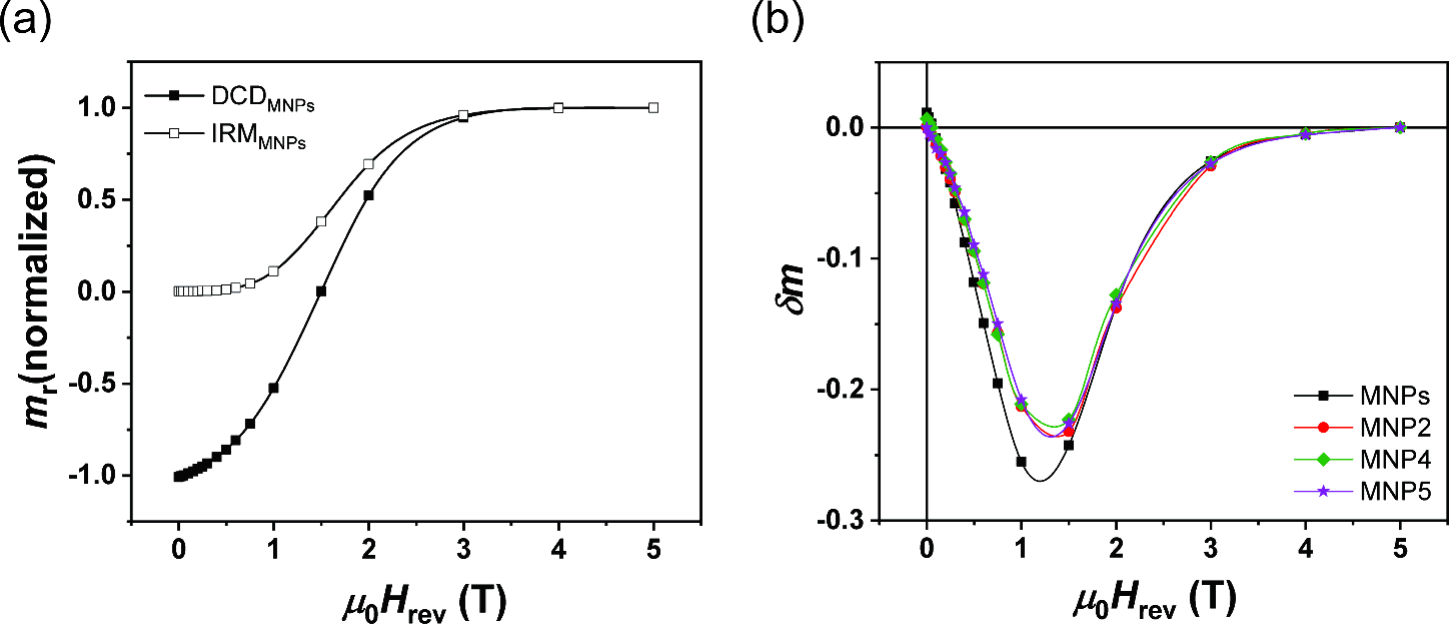
(a) IRM (isothermal remanent magnetization) and DCD (direct current demagnetization) remanent magnetization curves for bare CoFe_2_O_4_ nanoparticles. (b) *δm* plots for an assembly of interacting CoFe_2_O_4_ nanoparticles embedded in BCPs with different percentages (i.e., 19.4 wt % for MNP2, 4.6 wt % for MNP4, and 2.4 wt % for MNP5).

### Effect of Block Copolymer Structure on Nanoparticle Magnetic Properties

Similar *M*_r_/*M*_s_ were observed for MNPs (∼ 0.63) and MNP2 (∼ 0.68), suggesting the presence of uniaxial magnetic anisotropy.^55^ Reducing the amount of particles allows the preservation of the polymer lamellar structure (MNP4 and MNP5), and leads to an increase in the *M*_r_/*M*_s_ value up to ∼ 0.8, consistent with cubic anisotropy. These data reveal the key role of the matrix texture in modulating the magnetic anisotropy. As previously observed for 3D nanocrystal assemblies,^56^ iso-oriented MNPs aggregates,^57^ and MNPs embedded in mesoporous silica matrix,^58^ the possible explanation for the increase in *M*_r_/*M*_s_ can be the partial orientation of the nanoparticle easy axis. To further investigate the scenario of particle orientation, calculations based on the FC magnetization values at 5K were used to provide insight into the degree of alignment of the easy axes. The ratio of aligned (*M*_FC_^align^) to random (*M*_FC_) FC magnetization was calculated,^56^

2 $$\frac{M_{\mathrm{FC}}^{\mathrm{align}}}{M_{\mathrm{FC}}}=1+\alpha \left(3\cos^{2}\beta -1\right)$$

where β is the average angle between the easy magnetic axis and the applied magnetic field, and α is the fraction of nanoparticles with aligned easy axes. If all particles are randomly oriented, α = 0 and the ratio $\frac{M_{\mathrm{FC}}^{\mathrm{align}}}{M_{\mathrm{FC}}}=1$ , while the presence of a fraction of particles with oriented easy axes leads to a ratio ≠ 1. For our study, the obtained ratio values were 0.99, 5.6, and 5.7 for MNPs, MNP4 and MNP5, respectively. Despite the fact that this calculation provides only qualitative information, these deviations indicate a significant partial alignment (α > 0), with β → 0 (easy axes parallel to the field), driven by the effect of the lamellar BCPs matrix. As shown schematically in Figure 7, the fact that the mean interparticle interactions are comparable in all composites clearly indicates that the periodic lamellar BCP structure imposes directional confinement, which enhances the partial alignment of the easy axes and, as a consequence, the *M*_r_/*M*_s_ ratio increases. On the other hand, the disordered matrix in MNP2 promotes the random orientation of the easy axis, but reduces the dipolar interactions between the particles, which is manifested in the *M*_r_/*M*_s_ value (∼ 0.63). Thus, in the lamellar system, the matrix acts as a “soft template” that guides the magnetic nanoparticles into a more ordered structure. These results highlight the potential of our hybrid microparticles as a foundational platform for the development of magnetically responsive photonic systems. While the current architecture yields static structural coloration due to the rigid lamellar configuration formed during solvent evaporation, the observed magnetic characteristics – particularly the enhanced reduced remanent magnetization and the partial alignment of magnetic easy axes – suggest a promising degree of anisotropic ordering within the matrix. This inherent magnetic texture could be harnessed in future designs where the microparticles are embedded within deformable or elastomeric host materials. Such systems would enable field-induced reorientation or deformation of the photonic domains, paving the way for dynamic color changes in response to external magnetic stimuli. Furthermore, by modulating the mechanical properties and interfacial compatibility of the surrounding matrix, it may be possible to achieve reversible and tunable optical transitions. Overall, the integration of these structurally and magnetically engineered microparticles into responsive matrices holds significant promise for the realization of smart coatings, adaptive camouflage, soft robotics, and next-generation display technologies.

**Figure 7:**
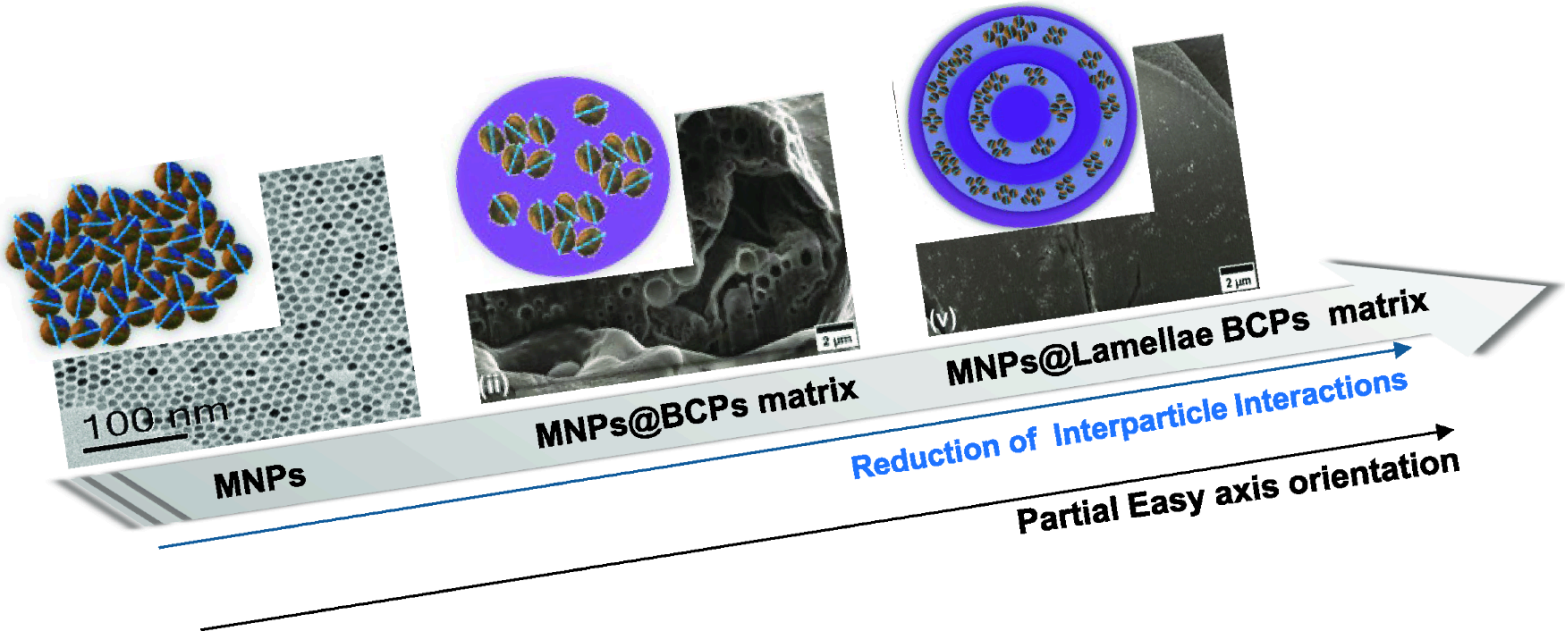
Schematic representation of nanoparticle arrangement and anisotropy easy axis orientation in bare particle MNPs, MNPs@BCP, and MNPs@lamellar BCP microparticles.

## Conclusion

This work presents a scalable strategy for the fabrication of hybrid photonic microparticles that integrate structural coloration with magnetic functionalities. The strategy involves confining the coassembly of poly­(styrene)-*b*-poly­(2-vinylpyridine) block copolymers and cobalt ferrite nanoparticles within emulsion droplets. This approach enables the creation of hierarchical onion-like architectures with alternating photonic layers selectively loaded with magnetic nanoparticles. This self-assembly process preserves the optical properties of the photonic structure while enabling tunable magnetic behavior, demonstrating a synergy between photonic and magnetic properties. We show that the selective localization of CoFe_2_O_4_ nanoparticles within the P2VP domains ensures the maintenance of a well-defined photonic bandgap up to a critical nanoparticle concentration. Beyond this threshold, the structural periodicity is disrupted, leading to a loss of optical response. Magnetic studies show that the embedding of 10 nm CoFe_2_O_4_ MNPs in a block copolymer matrix induces a partial orientation of their easy axes and a reduction of dipolar interactions. These results establish a paradigm for multifunctional materials in which nanoscale confinement can be exploited to coengineer structural and magnetic properties. The ability to integrate magnetically tunable photonic structures opens avenues for smart coatings, magneto-optical sensors, and next-generation responsive materials. Future efforts will focus on refining nanoparticle–polymer interactions to push the loading limits while maintaining structural integrity and extending this approach to alternative polymer systems for broader technological applications.

## Supplementary Material



## Data Availability

Data are openly available at Zenodo at 10.5281/zenodo.15119620.

## Notes

Figure S1, UV–vis absorption spectrum of cobalt ferrite nanoparticles; Figure S2, TEM micrographs of nanoparticle dispersions in block copolymer solutions; Figure S3, TGA and DTG profiles of BCP particles containing MNPs; Figure S4, FIB-SEM micrographs of BCP particles with varying MNP content; Figure S5, SAXS and USAXS spectra for MNP1-MNP5 samples; Figure S6, ZFC and FC magnetic characterization curves of MNP-embedded BCP particles; Figure S7, DCD and IRM magnetic curves for different MNP loadings; and Table S1, summary of hybrid particle compositions (PDF)
