# Supplementary material for: Co-assembly of Block Copolymers and Cobalt Ferrite Nanoparticles for Magnetic Material Design
Source: Chem Mater. 2025 Jul 10;37(14):5406–16. doi: 10.1021/acs.chemmater.5c01442 (PMC12288009; doi:10.1021/acs.chemmater.5c01442)
Supplement: Supplementary file 1 [file cm5c01442_si_001.pdf]

Supporting Information

Co-Assembly of Block Copolymers and Cobalt Ferrite Nanoparticles for Magnetic Material Design

Simone Bertucci[1], *Andrea Escher*[2], *Gianluca Bravetti*[1], *Jean Pierre Miranda Murillo*[2], *Gianluca Mazzotta*[3,4], *Sawssen Slimani*[2,5,6]*, *Stefano Alberti*[2], *Paola Lova*[2], *Davide Comoretto*[2], *Ullrich Steiner*[1,4], *Davide Peddis*[2,5,6], *Andrea Dodero*[1,2,4]*

[1] Adolphe Merkle Institute, University of Fribourg, Chemin des Verdiers 4, 1700 Fribourg, Switzerland

[2] Department of Chemistry and Industrial Chemistry, University of Genoa, Via Dodecaneso 31, 16146 Genoa, Italy

[3] Department of Chemistry, University of Fribourg, Chemin du Musée 9, 1700 Fribourg, Switzerland

[4] National Center of Competence in Research (NCCR) Bio-Inspired Materials, Chemin des Verdiers 4, 1700 Fribourg, Switzerland

[5] INSTM RU, nM2-Lab, University of Genoa, Via Dodecaneso 31, 16146 Genova, Italy

[6] nM2-Lab, CNR – Institute of Structure of Matter, 00015 Rome, Italy

Corresponding Authors

*Andrea Dodero, andrea.dodero@unifr.ch

*Sawssen Slimani, Sawssen.slimani@unige.it

Supporting Figures

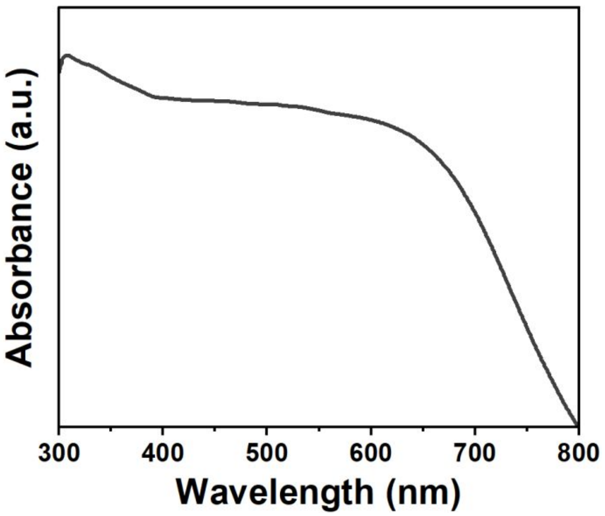

Figure S1. UV-vis absorption spectrum of cobalt ferrite nanoparticles dispersed in toluene at a concentration of 1 mg/mL. A broad absorption profile spanning the visible region is observed, consistent with the dark color of the NPs used.

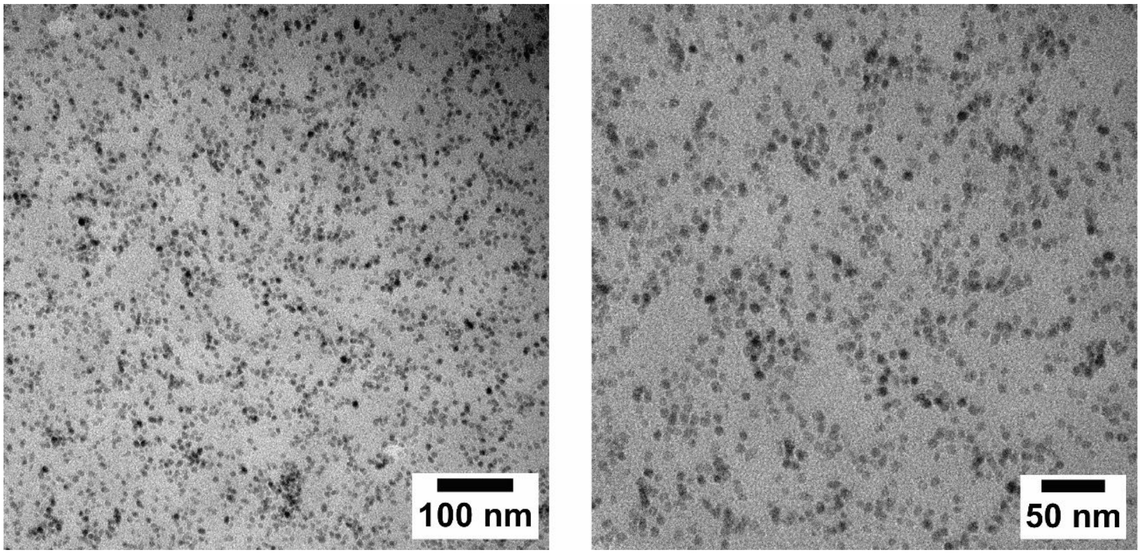

Figure S2. TEM micrographs at low and high magnification of a sample obtained by drying a nanoparticle dispersion (1 mg/mL) in block copolymer solution (10 mg/mL). The homogenous distribution of the nanoparticles indicated their optimal dispersion in the precursor solution.

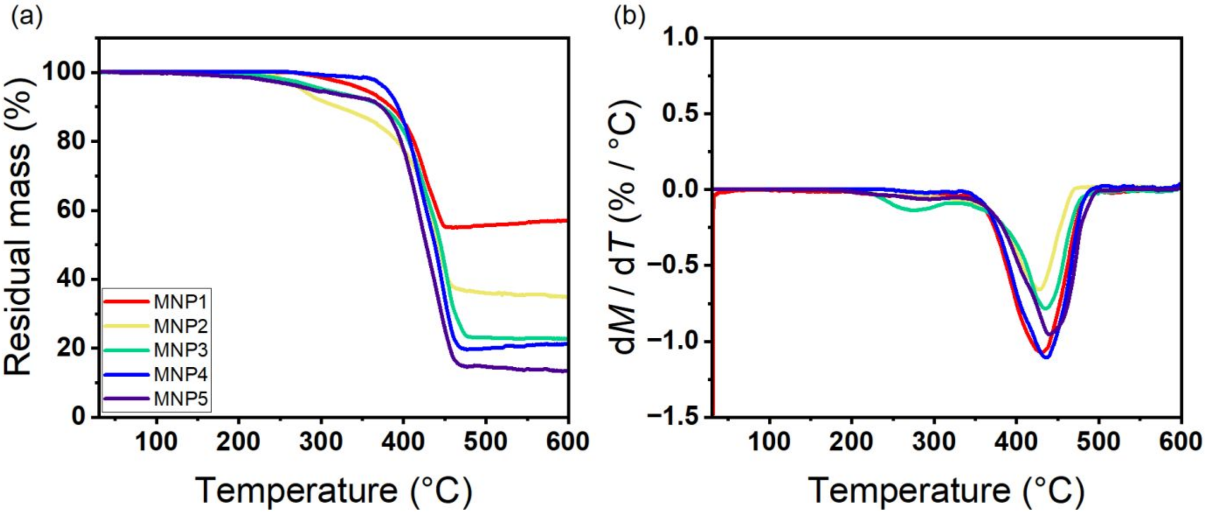

Figure S3. (a) TGA and (b) DTG profiles recorded in a nitrogen atmosphere for BCP particles containing different amounts of MNPs.

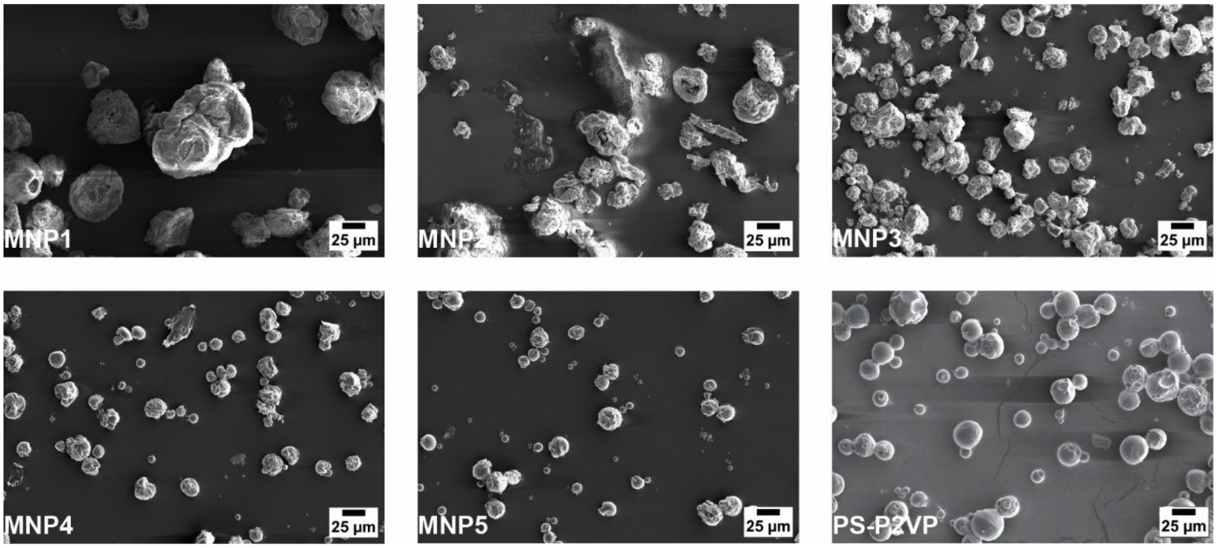

Figure S4. Top-view micrographs obtained by FIB-SEM for BCP particles containing different amounts of MNPs.

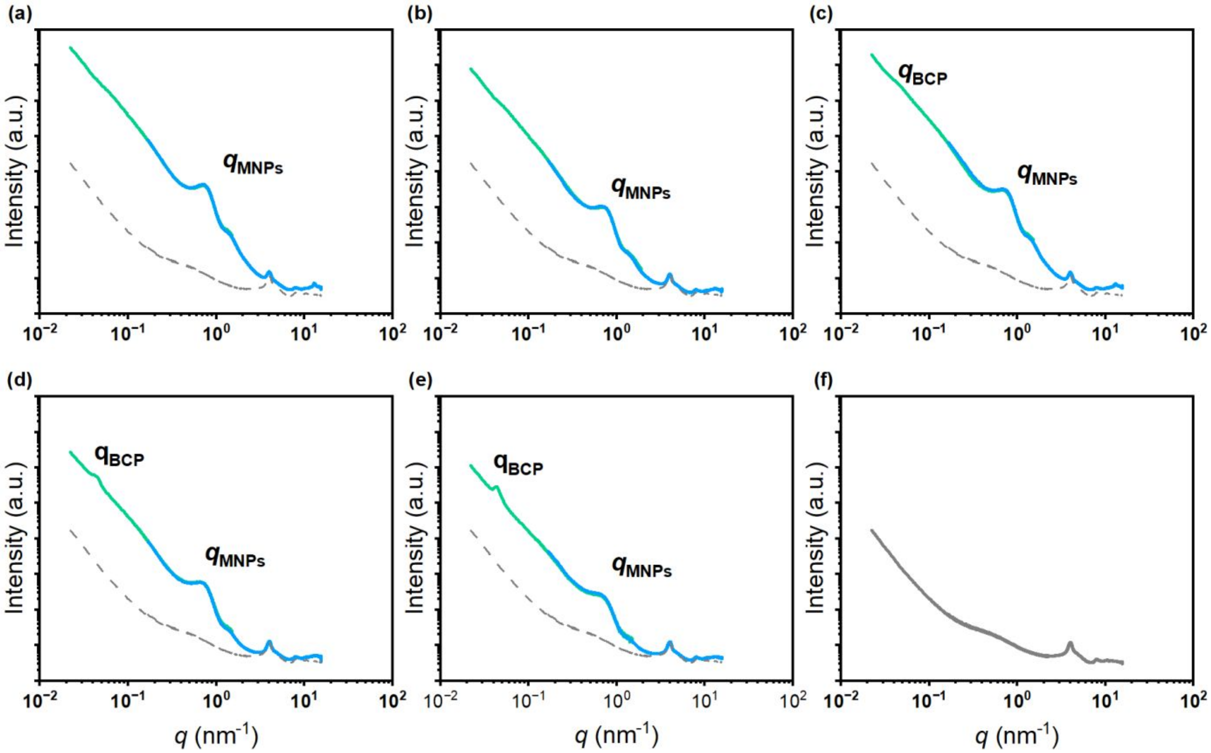

Figure S5. SAXS and USAXS spectra obtained for (a) MNP1, (b) MNP2, (c) MNP3, (d) MNP4, (e) MNP5 samples, and (f) the background.

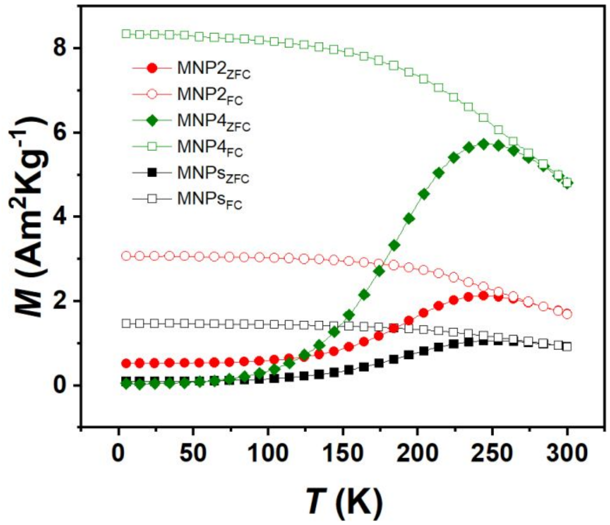

Figure S6. ZFC (full squares) and FC (empty squares) curves of MNPs nanoparticles embedded in BCP with different percentages (i.e., 19.4 wt% for MNP2, 4.6 wt% for MNP4, and 2.4 wt% for MNP5 samples).

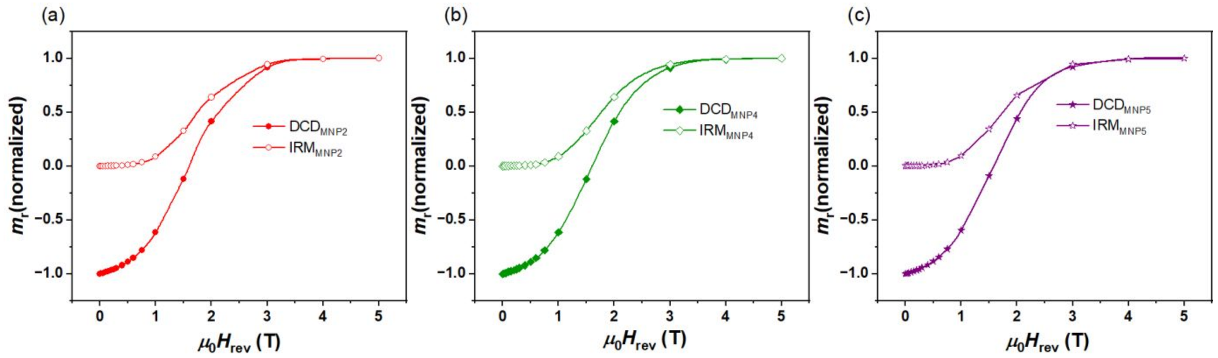

Figure S7. DCD (full symbols) and IRM (empty symbols) curves for different percentages of nanoparticles embedded in the BCP matrix, namely 19.4 wt% for MNP2, 4.6 wt% for MNP4, and 2.4 wt% for MNP5.

Supporting Tables

Table S1. Summary of the composition of the prepared hybrid particle samples.

Sample	BCP solution (mL)	BCP mass (mg)	MNP suspension (mL)	MNP mass (mg)	BCP nominal weight ratio (%)	MNP nominal weight ratio (%)	Residual mass at T= 600 ˚C (%)
MNP1	0.25	2.5	0.1	1.2	67.6	32.4	57
MNP2			0.05	0.6	80.6	19.4	35
MNP3			0.025	0.3	89.3	10.7	25
MNP4			0.01	0.12	95.4	4.6	21
MNP5			0.005	0.06	97.7	2.3	13
